# Solubility of Carbon Dioxide in Deep Eutectic Solvents Based on 3-Amino-1-Propanol and Tetraalkylammonium Salts at Low Pressure

**DOI:** 10.3390/ma14030594

**Published:** 2021-01-27

**Authors:** Iwona Cichowska-Kopczyńska, Dorota Warmińska, Bartosz Nowosielski

**Affiliations:** 1 Department of Process Engineering and Chemical Technology, Faculty of Chemistry, Gdańsk University of Technology, 80-233 Gdańsk, Poland; 2 Department of Physical Chemistry, Faculty of Chemistry, Gdańsk University of Technology, 80-233 Gdańsk, Poland; dorota.warminska@pg.edu.pl (D.W.); bartosz.nowosielski@pg.edu.pl (B.N.)

**Keywords:** carbon dioxide, capacity, solubility, equilibrium, deep eutectic solvent

## Abstract

Deep eutectic solvents (DESs) became an object of a great interest as an alternative to ionic liquids (ILs) and commonly used in CO_2_ capture amine solutions. In the present study, five different DESs based on 3-amino-1-propanol as physical-chemical CO_2_ absorbents were used. The composition was chosen in order to estimate the effects of hydrogen bond acceptor:hydrogen bond donor (HBA:HBD) molar ratio, anion type and length of alkyl chain of composing salt. The Fourier Transform Infrared (FTIR) spectroscopy was used to confirm chemical reaction. The solubility of CO_2_ was measured at low pressures up to 170 kPa at the temperature range of 293–318 K. Viscosity, polarity and Kamlet–Taft parameters were determined in order to estimate the dependences of the parameters and the CO_2_ capacity. CO_2_ uptake was observed to improve with decreasing molar ratio of hydrogen bond donor. Comparing the CO_2_ capacity of [TBAC]-based DESs, at the approximate pressure of 50 kPa, it was observed that the capacity increased in the following order of molar ratios—1:8 < 1:6 < 1:4 and a decrease in molar ratio from 1:8 to 1:4 resulted in about a 100% increase of capacity. Compared to [TBAC][AP] DESs, the [TEAC][AP] 1:4 and [TBAB][AP] 1:4 exhibited higher CO_2_ uptake, though the best results were obtained for [TBAB][AP].

## 1. Introduction

Carbon dioxide is considered as a key gas in the enhancement of global warming and climate change. Increasing global CO_2_ emissions forced researchers to comprehensively investigate its capture and storage technologies that should be efficient and economic. Approximately 40% of total emission of carbon dioxide comes from fossil fuel-based power plants, making them perfect targets for immediate CO_2_ reductions [1]. Therefore, removal of CO_2_ from exhaust streams is of particular importance in terms of atmosphere protection. Several methods of CO_2_ capture were extensively studied over the last decades. The most widely used technology is chemical absorption involving aqueous amino acid salts, ammonia and alkanolamines. It has been established so far that aqueous amino acid salts from neutralization of amino acids with an organic base such as an amine showed better CO_2_ absorption capacity than amino acid salts formed by neutralization of amino acids with an inorganic base such as potassium hydroxide [2,3]. 

The research performed by Bai and Yeh showed that the maximum CO_2_ removal efficiency by an ammonia absorber can reach 99% and the CO_2_ loading capacity can approach 1.20 g CO_2_/g NH_3_ [4].

However, because of smaller toxicity compared to ammonia and higher efficiency compared to amino acids, the most often used in industrial CO_2_ separation are alkanolamines [5,6]. Commercially, a 30% aqueous solution of monoethanolamine (MEA), as the most effective and cheapest, is used [7]. A major problem in the usage of alkanolamines for CO_2_ absorption is equipment corrosion and cost of regeneration [8]. 

In the last decades much attention was given to the ionic liquids (ILs), considered as a “greener” replacement for amines, that present many advantages. They have negligible vapor pressure, which is of particular importance from the point of view of industrial applications, they are non-flammable, present high thermal stability, can be easily tuned for a specific application and are able to absorb a wide range of chemicals, including CO_2_ [9,10]. The majority of research reports concludes that ILs absorb carbon dioxide physically. Physical absorption results in a low cost of solvent regeneration, but it strongly limits their applications in CO_2_ capture at low partial pressure of post-combustion flue gas [11]. Industrial use of ionic liquids is very poor, mainly because of the high price, which excludes them from bulk applications. Moreover, there are data exhibiting their toxicity and non-biodegradability [12,13].

Therefore, to overcome the shortcomings of ILs, deep eutectic solvents (DESs) started to be considered as new absorbents for CO_2_ capture. Similar to ILs, deep eutectic solvents are practically non-volatile and non-flammable, exhibit high thermal and electro-chemical stability, but are definitely cheaper, less toxic and often biodegradable, which makes them better solvents for carbon dioxide absorption [14]. They are obtained by simply mixing two substances, a hydrogen bond donor and acceptor, resulting in an eutectic mixture with melting point lower than that of each of the two components of DES. Physical properties of DESs are tunable and can be controlled by changing the composition and proportions of the components to obtain properties essential for a particular application [15]. 

The carbon dioxide absorption in deep eutectic solvents can be physical, chemical or mixed depending on the compounds used to obtain DES [16]. The majority of works is focused on deep eutectic solvents in which physical absorption of carbon dioxide occurs, mainly based on choline chloride (ChCl) acting as a hydrogen bond acceptor (HBA) and urea, glycols and carboxylic acids as hydrogen bond donors (HBDs) [14]. These systems are characterized by CO_2_ capacity similar to that of ILs and by lower CO_2_ solubility than aqueous solutions of hydroxyamines (e.g., at 298 K and 10 bar in ChCl:TEG CO_2_ solubility is equal to 2.0 wt.% while in aqueous 30% MEA it is 11.95 wt.%) [17,18,19,20].

To improve carbon dioxide absorption capacity, deep eutectic solvents based on alkanolamines or amines as HBDs were introduced. Ali et al. [19] reported series of DESs which were composed of choline chloride, methyltriphenylphosphonium bromide (MTPPB) and tetrabutylammonium-bromide (TBAB) as HBAs and ethanolamine (MEA), diethanolamine (DEA) and triethanolamine (TEA) as HBDs. The obtained results show that solubility of CO_2_ in alkanolamine-based DESs is higher than for glycol-based. Additionally, it was shown that for MTPPB-based DESs, increasing the fraction of MEA results in the decrease of CO_2_ solubility. Adeyemi et al. [21] reported ChCl-based DESs coupled with MEA, DEA and methyldiethanolamine (MDEA) at three different molar ratios of 1:6, 1:8 and 1:10. The authors observed that the solubility of carbon dioxide in amine-based DESs is much higher (approx. 0.3 g of CO_2_/g of ChCl-MEA 1:8) than for 30 wt.% MEA solution (approx. 0.1 g of CO_2_/g of solvent). The highest carbon dioxide absorption was reported for ChCl-MEA and the lowest was for ChCl-MDEA. Additionally, they concluded that as a molar ratio of amine increases, the solubility of CO_2_ also increases. Haider et al. [22] described the influence of HBAs (ChCl and TBAB) on carbon dioxide absorption in ethylene glycol (EG), diethylene glycol (DEG), MDEA and DEA-based DESs. The authors reported that the highest absorption was obtained in TBAB-based DESs which was explained due to increase in free volume in TBAB-based DESs in comparison to ChCl-based DESs. Trivedi et al. [15] described novel deep eutectic solvents based on various amine hydrochlorides, namely—(MEACl), (TEACl), urea hydrochloride (UCl) and thioacetamide hydrochloride (TAECl), as HBAs and ethylenediamine (EDA) as an HBD. High gravimetric uptakes of carbon dioxide were reported with the greatest value for MEACl-EDA (mole ratio 1:3) being 31.8 wt.% 

Herein, a carbon dioxide absorption capacity of previously described by our group [23] 3-amino-1-propanol (AP)-based deep eutectic solvents is reported. AP was mixed with tetrabutylammonium bromide (TBAB) or tetrabutylammonium chloride (TBAC) or tetraethylammonium chloride (TEAC), which act as hydrogen bond acceptors to form DESs. The experiments were conducted at temperature range from 293.15 to 318.15 K at low pressures of CO_2_. Additionally, the effect of mole ratio of TBAC and AP on CO_2_ capacity was examined.

## 2. Materials and Methods 

**Figure 1:**
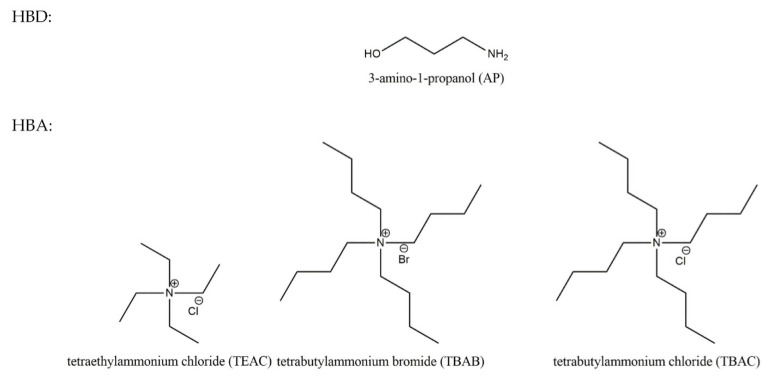
Structures of compounds used for deep eutectic solvents (DES) preparation.

The chemicals used in this study, tetrabutylammonium chloride, tetrabutylammonium bromide, tetraethylammonium chloride and 3-amino-1-propanol were purchased from Sigma-Aldrich (Saint Louis, MO, USA) and were used as received, only TBAC was purified by double crystallization from acetone by adding diethyl ether. Prior to synthesis the salts were dried in a vacuum oven (Thermo Scientific, Waltham, MA, USA), at 323 K for 48 h and 298.15 K for 3 days, respectively for TBAB and both TBAC and TEAC. Corresponding data are presented in Table 1. Figure 1 shows the structures of compounds used for DES preparation. Carbon dioxide was supplied by Oxygen S.C. Gdansk (Gdansk, Poland) with purity grade 5.0.

### 2.1. Synthesis of Deep Eutectic Solvents

The absorbents were prepared by mixing TBAB, TBAC and TEAC with 3-amino-1-propanol at the molar ratios of 1:4, 1:6 and 1:8. Weighting was done with an electronic balance (Mettler Toledo, Columbus, OH, USA) with the precision of 0.1 mg. The HBD and HBA were mixed at 353.15 K for 1 h with a magnetic stirrer until homogeneous liquid was obtained. Resulting DESs were stored in gas-tight bottles at room temperature.

Before experiments DESs were dried in a vacuum dryer (Thermo Scientific, Waltham, MA, USA) at 343.15 K for 48 h. Water content was then determined using Karl Fischer titration method using 831 KF Coulometer apparatus from (Metrohm, Herisau, Switzerland). The contents of H_2_O were lower than the 0.002 weight fraction.

### 2.2. Properties of Deep Eutectic Solvents

#### 2.2.1. Viscosity Measurements

Viscosity was measured at temperatures ranging from 298.15 to 318.15 K and at atmospheric pressure. The LVDV-III Programmable Rheometer (Searle cone-plate viscometer; Brookfield Engineering Laboratory, Middleboro, MA, USA), controlled by a computer, was used to carry out measurements. To control the temperature of the samples within ±0.01 K accuracy, a thermostatic water bath (PolyScience 9106, Warrington, PA, USA) was used. CP-40 cone (angle 0.8°, radius 2.4 cm) and 0.5 ml deep eutectic solvent were used. The ratio of shear stress (N/m^2^) and shear rate (1/s) at 10 points was used to obtain flow curves which were used to calculate the dynamic viscosity. The viscometer was adjusted with certified viscosity standard N100 and S3 provided by Cannon at 298.15 ± 0.01 K. The standard uncertainty of viscosity measurement was better than 1%. 

#### 2.2.2. Polarity and Kamlet-Taft Parameters

Ultraviolet visible (UV-vis) absorption spectra of three solvatochromic dyes (Reichardt’s dye, N,N-diethyl-4-nitro-aniline, and 4-nitroaniline) dissolved in DESs were taken in a quartz cell with a light path length of 10 mm on a Thermo Scientific™ Evolution™ 300 spectrophotometer (Thermo Scientific, Waltham, MA, USA). Individual stock solutions of Reichardt’s dye, N,N-diethyl-4-nitroaniline and 4-nitroaniline were prepared in methanol. To prepare a given dye/DES solution, the appropriate amount of the dye stock solution was micropipetted into the Eppendorf flask and methanol was evaporated under a vacuum. The DES was then added and stirred until complete dissolution of the dye was achieved. Next, the resultant solution was transferred into the quartz cuvette, and the UV-vis spectra were recorded at room temperature.

### 2.3. Infrared Spectroscopy Measurements

To characterize the DESs before and after saturation with CO_2_ an attenuated total reflectance Fourier transforms infrared spectroscopy (ATR-FTIR) was used. For this purpose, the Nicolet 8700 spectrometer (Thermo Electron Co., Waltham, MA, USA) was applied. Employment of an attenuated total reflection (ATR) was needed because of the strong ν(OH)/ν(NH_2_) stretching vibrations of AP. The Specac Golden Gate single reflection accessory with a diamond crystal (45°) mounted in a heated tungsten carbide disc was used. The temperature of the sample was kept at 298.15 ± 0.1 K by circulating thermostated water from a Julabo F12 thermostat. The spectrometer was purged with dry nitrogen (Oxygen Gdansk, Gdansk, Poland). Prior to each experiment, the sample spectra were ratioed against a background spectrum collected for an empty, dry cell. For each spectrum, 256 scans were performed with a selected resolution of 2.0 cm^−1^. Spectra acquisition was controlled by OMNIC 7.3 software package (version 7.3, Thermo Scientific, Waltham, MA, USA). The spectra were analyzed using GRAMS/32 software (version 4.01A, Galactic Industries Corp., Salem, NH, USA).

### 2.4. CO_2_ Solubility

**Figure 2:**
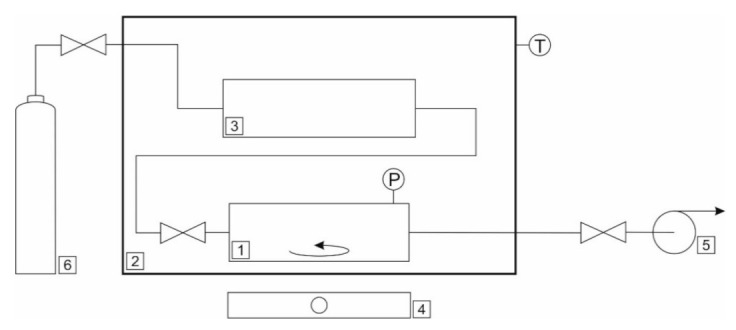
Schematic diagram of the equipment used for CO_2_ solubility measurement (1—equilibrium cell, 2—thermostat with temperature sensor, 3—gas container, 4—magnetic stirrer, 5—vacuum pump, 6—gas reservoir).

Schematic diagram of the equipment used for CO_2_ solubility measurement is presented in Figure 2. The experimental setup consisted of a stainless steel equilibrium chamber (1) equipped with a water jacket connected to the water bath temperature controller (PolyScience 9106, Warrington, PA, USA) (2), to a gas container (3) and to a vacuum pump (5). The equilibrium cell had a magnetic mixer (IKA, Staufen im Breisgau, Germany), (4) in order to mix the fluids and to provide that equilibrium state was reached faster. The volume of the cell was 7.8 ± 0.01 cm^3^. A volume of about 1 cm^3^ of DES was injected to the cell. The actual amount was determined gravimetrically using RADWAG analytical balance. The cell, absorbent and gas container were degassed with the vacuum pump (VALUE, Wenling, China). The gas container was then filled with CO_2_ from a gas reservoir (6). Subsequently, both the equilibrium cell and the gas container were thermostated at a given temperature. The experimental uncertainty for the temperature was ±0.1 K. When the temperature of fluids was equal and stable, the gas was injected to the equilibrium cell and a starting pressure was recorded. The pressure was measured by an Aplisens PC-28 transducer. As the CO_2_ was absorbed, the pressure in the cell decreased. When the equilibrium was reached, meaning the pressure in the cell did not change, the equilibrium pressure was recorded. The amount of CO_2_ absorbed was calculated using Equation (1).

## 3. Results

The initial amount of solute (*n*_0_) was obtained from Equation (1):

(1) $$n_{0}=\frac{p_{0}V}{Z_{2}RT}$$

where *V* is the volume of the gas phase in the measuring chamber, *p*_0_ is the initial gas pressure, *R* is the universal gas constant, *T*—temperature, *Z*_2_—is the compressibility factor expressed with Equation (2):

(2) $$Z_{2}=1+\frac{pB_{22}}{RT}$$

where *B_22_* is the second virial coefficient for pure CO_2_. The quantity of solute present in the gas phase was calculated with Equation (3):

(3) $$n_{1}=\frac{p_{1}V}{Z_{2}RT}$$

The amount of CO_2_ dissolved in the solvent is represented by the difference in CO_2_ mole values and was calculated by:

(4) $$n_{l}=\;n_{0}-n_{1}$$

At zero time the gas was put in contact with eutectic solvent and after that the decrease of pressure was observed due to the solubilization of the CO_2_ in DES. At low pressures, the equilibrium was reached within 24 h, though the higher pressure the more time was needed for equilibration, reaching 14 days. The reason was increasing viscosity and resulting diffusion resistance. Higher viscosity creates several effects, like slower diffusion of CO_2_ to the bulk solvent, inhibition of movement of CO_2_-loaded solvent to the bulk liquid and hindrance in diffusion of free solvent to the gas–liquid interface. Moreover, the higher viscosity the slower is the heat transfer. Therefore, when viscosity decreases due to increase of temperature, the equilibrium state is obtained faster. For that reason the more CO_2_ was added to the cell, the longer the time of equilibration. Temperature increase resulted in an increase in pressure caused by temperature change and by the change in solubility. After the equilibrium was reached at 293 K and the temperature was raised, it took a maximum of 2 h to reach the equilibrium again. The increase in temperature results first of all in lower viscosity and second of all lower gas solubility, therefore during the experiment the increase in equilibrium pressure was observed when the temperature raised. The time of equilibration was very short and that indicated easy regeneration of DESs used in the study. 

Viscosity plays a key role in mass and heat transfer and affects the economy of the process, operation parameters and the design of the unit itself. Among examined DESs [TEAC][AP] showed the lowest viscosity, and a higher one was observed for [TBAC][AP] and the highest for [TBAB][AP]. This affected the overall solvent transport properties, i.e., kinetics of absorption and the time of equilibration.

The observed CO_2_ uptake values were relatively high for all DESs, as 3-amino-1-propanol exhibits a high reaction rate with CO_2_ and therefore even at low pressures gives good results.

**Figure 3:**
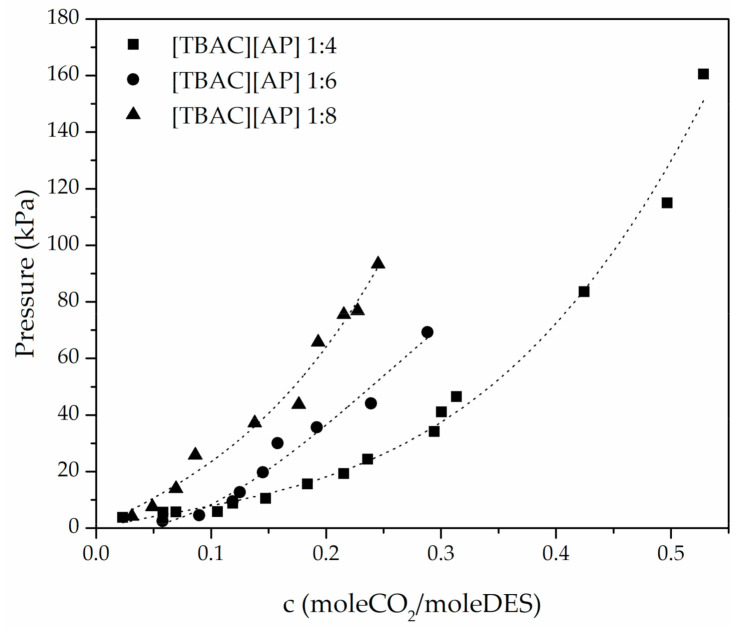
CO_2_ uptake by DES with different molar ratio.

The shape of the absorption curve is polynomial at the pressure range studied within this research (Figure 3). This is mainly due to the chemical reaction that takes place and is in agreement with the research performed by Dong et al. who obtained similar results for the solution of 1-amino-1-propanol. The authors observed an inflection point above 100 kPa [24]. 

The values obtained for [TBAB][AP] 1:4 were close to that reported by Shukla et al. [25], around 0.51 moleCO_2_/moleDES at atmospheric pressure. CO_2_ capture in DESs can be tailored by varying the molar ratio of the hydrogen bond donor, changing the strength of the hydrogen bond between components or by affecting the physical absorption in a form of the size of the HBA. Due to the chemisorption occurring in DESs used in this study, the CO_2_ uptake was much higher than in DESs as physical absorbents, such as for example tetrabutylammonium bromide coupled with methyldiethanol that was studied by Haider and gave a capacity of 0.29 moleCO_2_/mole solvent at high pressure of 1 MPa and 303.15 K [22].

**Figure 4:**
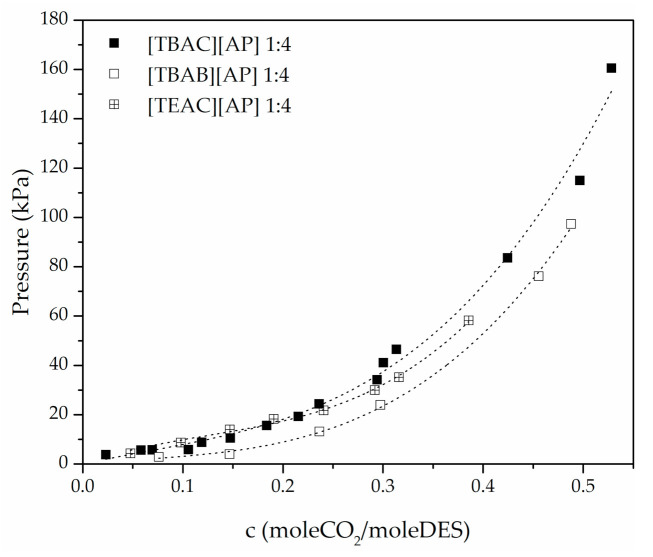
CO_2_ uptake in different DESs of 1:4 molar ratio.

For the example of [TBAC][AP], CO_2_ uptake was observed to improve with decreasing molar ratio of hydrogen bond donor (3-amino-1-propanol), as shown in Figure 3. The CO_2_ absorption at the approximate pressure in TBAC-based DESs increased about 100% from 1:8 to 1:4, whereas 1:6 was between. As presented in Figure 4, compared to [TBAC][AP] DESs, the [TEAC][AP] 1:4 and [TBAB][AP] 1:4 exhibited higher CO_2_ uptake, though the best results were obtained for [TBAB][AP].

Literature describes the cases where the CO_2_ absorption trend varies depending on the HBD and HBA ratio. For example, Zhang et al. conducted research on absorption in tetraethylenetetramine chloride [TETEA.Cl] and ethylene glycol [EG] or diethylene glycol [DG] and discovered that the CO_2_ capacity for [TETEA.Cl][DG] increases from 1:1 to 1:2 and then decreases up to 1:7 and for [TETEA.Cl][EG] increases from 1:1 to 1:3 and further decreases [26]. However, for MEA in the role of HBD and choline chloride, tetraethylammonium chloride and tetramethylammonium chloride as HBA, Zhuo et al. reported increasing CO_2_ absorption from 1:2 up to 1:6. Surprisingly, the authors reported also increase of CO_2_ capacity when increasing temperature, suggesting that the reaction is endothermic [27]. Furthermore, Adeyemi et al. reported increasing CO_2_ capacity of [ChCl][MEA] and [ChCl][DEA] from 1:6 to 1:10 [21].

The comparison of CO_2_ solubility in the studied DESs indicates that the CO_2_ solubility deceases with increasing alkyl chain length of the hydrogen bond acceptor. Moreover, exchange of the anion of the composing salt from Br^−^ to Cl^−^ results in a significant decrease in solubility. 

**Figure 5:**
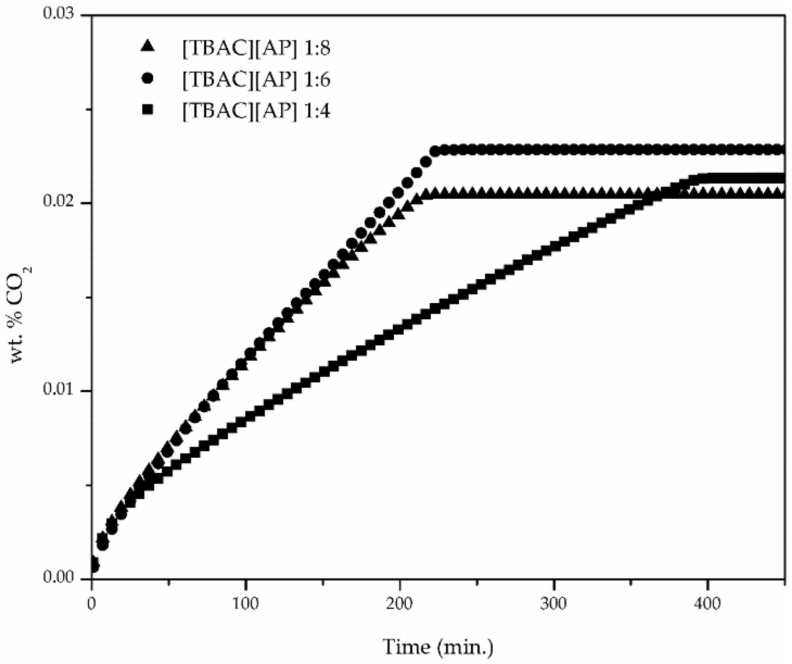
Time-dependent CO_2_ absorption of DESs with different molar ratio of HBA and HBD.

**Figure 6:**
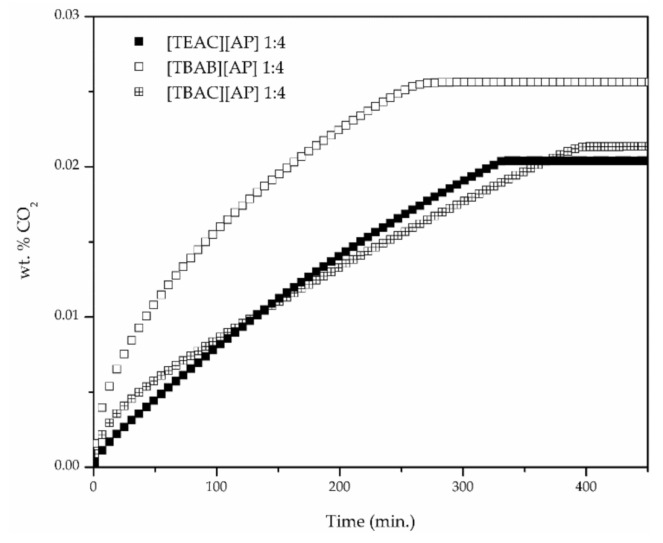
Time-dependent CO_2_ absorption of DESs with different hydrogen bond acceptors (HBAs).

The rate of CO_2_ absorption for DESs with different molar ratio of HBA:HBD is shown in Figure 5. The graph applies to the first measuring point for each liquid, however, at different CO_2_ pressures, the trend does not coincide with the total CO_2_ capacity of the DESs. In the first 20 min of absorption after the CO_2_ was introduced to the cell, the rate of the absorption was similar for all three molar ratios. Then, there was another approximately 20 min when the rate of absorption was in the following order—1:8 > 1:6 > 1:4, which was in agreement with the initial viscosity of the pure DESs (Table 2). After that, the rate of the process dramatically decreased in the case of 1:4 DES whereas the 1:6 and 1:8 solutions stayed close. A temperature increase during the stage of high absorption rate was not observed, however the temperature inside the cell was not directly measured, but the measuring cell was kept in the thermostated water bath of a high volume in comparison to the volume of the cell. Taking into account the highly exothermic reaction and its effect on viscosity and energy of the system, it is likely that thermal effects play an important role in that phenomena and it is worth evaluating together with the changes of viscosity during the absorption. In comparison to different HBAs with the same HBA:HBD ratio, the absorption rate at the initial stage was the highest for DES with the highest viscosity, and the lowest for the [TEAC][AP], which has the lowest viscosity (Figure 6). Until equilibrium was reached, the rate for DESs based on the chloride salts stayed close, whereas for [TBAB][AP] the rate was much higher. 

In general, the time of reaching equilibrium was short at low pressures, however increasing CO_2_ pressure resulted in a very long time of equilibration. This is attributed to the increasing viscosity [28] upon CO_2_ absorption that causes higher resistance of diffusion and hindrance in migration of CO_2_ molecules [29,30]. The observed increase of viscosity was very high. Idris et al. performed a study on viscosity of 3-amino-1-propanol water solution unloaded and loaded with CO_2_ and they reported about a 73% viscosity increase of 0.5 wt.% [AP] solution at 0.058 mole fraction of CO_2_ (from 7.58 to 13.14 mPas) at 298.15 K [28]. Trivedi et al. also reported a dramatic increase in viscosity of DESs, for example for [MEACl][EDA], after 2.5 min of saturation the viscosity increased from 21.6 to 3995 cP at 30 °C [30]. 

Detailed results and analysis of physicochemical properties were provided elsewhere by Nowosielski et al. [23]. Within this paper, viscosity at 298.15 K and at atmospheric pressure (*p* = 0.1 MPa) and solvatochromic parameters at room temperature are presented. As the 3-amino-1-propanol is liquid at room temperature with viscosity of 30.43 mPa·s [28], while the HBA is not, the viscosity of all DESs is higher than that of pure [AP] and decreases with the increase of HBD to HBA ratio. As can be seen from Table 2, the experimental values of solvatochromic parameters were in good agreement with values found in the literature.

**Figure 7:**
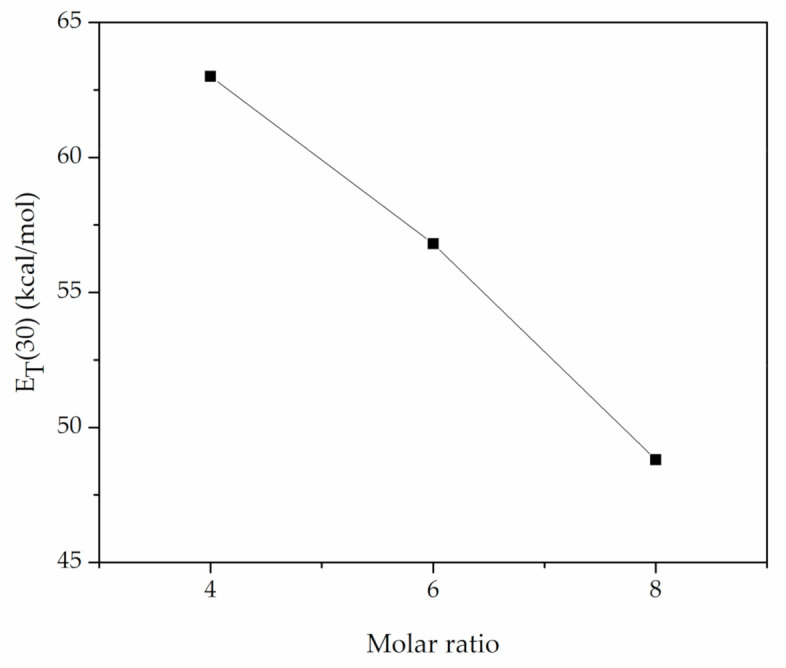
Effect of HBD:HBA ratio on E_T_(30).

**Figure 8:**
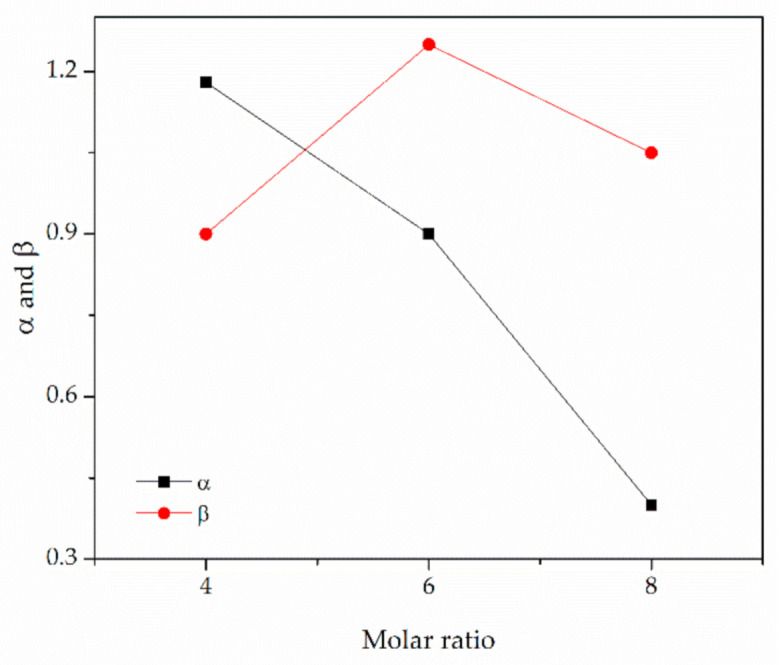
Effect of HBD:HBA ratio on acidity and basicity.

Taking into account E_T_(30), it was observed that the higher electronic transition energy results in higher CO_2_ solubility (Figure 7). Moreover, as described by Shukla et al. [31] the relative value of α-β describes the nature of interactions between DESs and CO_2_. The closer the strengths of the donor and acceptor are, the more favored the synergistic action and the higher the CO_2_ capacity observed (Figure 8). This phenomenon can be considered in the prediction of the optimum HBA:HBD ratio for a specific DES, though it should not be used for comparison of DESs with different types of composing agents. This was confirmed by Shukla and Mikkola [25] who studied [TBAB][AP] and [HMIMCl][AP] and observed an increase in CO_2_ capacity with increasing [AP] content, due to the favored synergistic effect. Our results confirm that the CO_2_ uptake in DESs depends on the equilibrium between α and β that describes the intermolecular interactions between the HBD and HBA.

The solubility over the series of DESs used in this study varies as follows—[TBAB][AP] > [TEAC][AP] > [TBAC][AP]. With regard to the synergistic effect, [TEAC][AP] should have the best solubility, but apart from the basicity and acidity of DESs components, the solubility is influenced by the free volume and the strength of hydrogen interactions between the components. Solubility increases with increasing free volume and decreases with increasing strength of hydrogen interactions. Apparently, the superimposition of all these effects determines the ability of the DESs to dissolve carbon dioxide. The synergistic effect is most evident in the case of [TEAC], while in the case of [TBAB] the free volume favors the CO_2_ absorption. Moreover, the relative special position of the functional groups in the molecule is essential. According to Shukla et al., the presence of AP with an acidic proton –OH is crucial in CO_2_ capture. As the –OH group is at the α position to NH_2_ it stabilizes CO_2_ at the carbamic acid form [25]. 

It has been established that in deep eutectic solvents based on alkanolamines, chemisorption of carbon dioxide occurs [22,32]. The reaction of CO_2_ and DESs results from the content of primary alkanolamine AP and the reactions of its functional groups −NH_2_ and −OH:

−NH_2_ + CO_2_ = −NHCOO^−^H^+^

−OH + CO_2_ = −OCOO^−^H^+^

**Figure 9:**
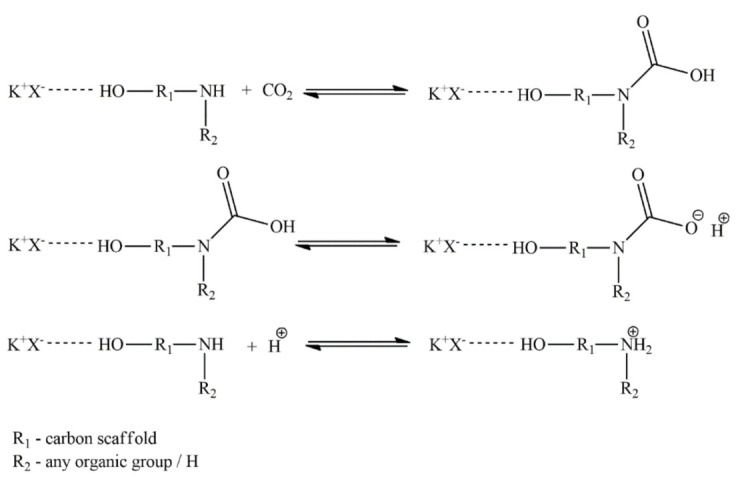
Scheme of alkanolamine and CO_2_ reactions.

However, according to Astarita and other authors [33,34], the latter reaction is negligible due to the conditions needed and the main product of the reaction is carbamate. The molecule 3-amine-1-propanol follows the mechanism presented in Figure 9. As can be seen, due to the reaction, carbamate is formed [30]. 

**Figure 10:**
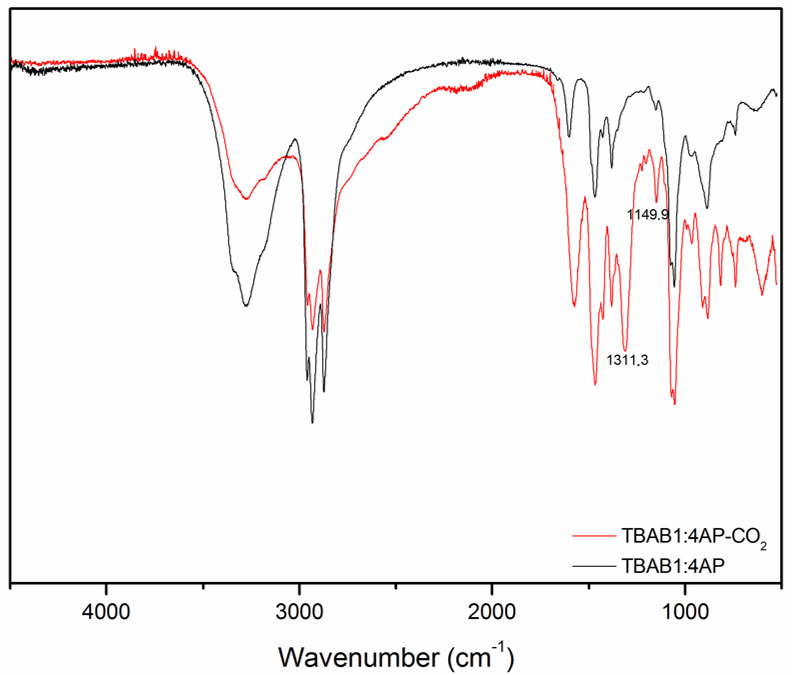
Spectroscopic analysis of [TBAC][AP] 1:4 before and after CO_2_ absorption.

Figure 10 presents the FTIR spectra of [TBAC][AP] 1:4 before and after CO_2_ absorption. As can be seen, after carbon dioxide absorption in the [TBAC][AP] 1:4, new peaks in the fingerprint region at 1311 and 1150 cm^−1^ appear. It is clear that they belong to N-C=O(O) and C-N stretching in formed carbamate due to reaction of CO_2_ and alkanolamine [32]. Similar new peaks were observed in [TBAB][AP] and [TEAC][AP] spectra, however the positions of the ν(N-C=O(O)) bands become slightly red-shifted while the frequency of C-N stretching is practically constant for all DESs. The frequency of N-C=O(O) band changes is in the order [TBAB][AP] > [TBAC][AP] > [TEAC][AP]. Thus, it depends both on an anion and a cation of the hydrogen bond donor. 

The reaction results in the temperature rise at the interfacial film. According to Camacho [34] it is dependent on the AP concentration and can be significant at certain conditions and can cause evaporation to the gas phase.

According to data provided by Benamor et al., stability of carbamates based on 3-amino-1-propanol is higher than of other amines like MEA or DEA. The authors also reported that AP carbamate is less sensitive to the temperature than other two. Still, the equilibrium constant for 3-amino-1-propanol carbamate formation was decreasing as the temperature increased, as the reaction is exothermal [35].

## 4. Conclusions

In the present study, the CO_2_ solubility in five different DESs at several temperature points and varying pressure was investigated. Viscosity and polarity data were also reported. It was found that CO_2_ uptake was improving with decreasing molar ratio of 3-amino-1-propanol. For [TBAC]-based DESs, the CO_2_ capacity at the approximate pressure was in the following order—1:8 > 1:6 > 1:4. Considering the impact of the anion of tetrabutylammonium salt, it was found that DES with a bromide anion, namely [TBAB][AP] 1:4 exhibited higher CO_2_ uptake than chloride-based DES. In the estimation of absorption kinetics, several stages with different characteristics were observed, and the rate of CO_2_ absorption for DESs with different molar ratios of HBA:HBD was found to be similar for all three molar ratios at the initial stage, then there was another stage when the rate of absorption was in the following order—1:8>1:6>1:4, which was in agreement with the initial viscosity of the pure DESs, after that the rate of the process dramatically decreased in case of 1:4 DES whereas that of 1:6 and 1:8 stayed close. It was also observed that the rate of absorption was dependent on the anion of the composing salt. The evaluation of such behavior should be an object of interest for another study combining the changes of viscosity and thermal micro effects during the absorption.

## Figures and Tables

**Table 1:** Sources and mass fraction purity of used chemicals.

Chemical Name	Source	CAS Number	Purity/Mass Fraction ^a^
3-amino-1-propanol [AP]	Sigma Aldrich	157-87-6	0.99
Tetrabutylammonium bromide [TBAB]	Sigma Aldrich	1643-19-2	≥0.99
Tetrabutylammonium chloride [TBAC]	Sigma Aldrich	1112-67-0	≥0.99
Tetraethylammonium chloride [TEAC]	Sigma Aldrich	56-34-8	≥0.99

^a^—according to the supplier.

**Table 2:** Thermophysical properties of the DES at p = 0.1 MPa, 298.15 K and comparison with literature data.

DES	Molar Ratio	ƞ/(mPa.s)	E_T_(30)/(kcal/mol)	π	α	β	|α−β|
TBAB-AP	1:4	84.69	48.7 (48.5 ^a^)	1.07 (1.02 ^a^)	0.37 (0.39 ^a^)	1.00 (0.90 ^a^)	0.63
TEAC-AP	1:4	42.37	56.7	1.10	0.87	0.92	0.05
TBAC-AP	1:4	77.36	63.0	1.13	1.18	0.90	0.28
TBAC-AP	1:6	53.86	56.8	1.02	0.90	1.25	0.34
TBAC-AP	1:8	46.14	48.8	1.04	0.40	1.05	0.65

^a^ Experimental data reported by Shukla and Mikkola [25].

## Data Availability

Data available on request due to privacy restrictions.
